# Correction: Recombinant Lloviu virus as a tool to study viral replication and host responses

**DOI:** 10.1371/journal.ppat.1010659

**Published:** 2022-06-24

**Authors:** Adam J. Hume, Baylee Heiden, Judith Olejnik, Ellen L. Suder, Stephen Ross, Whitney A. Scoon, Esther Bullitt, Maria Ericsson, Mitchell R. White, Jacquelyn Turcinovic, Tran T. N. Thao, Ryan M. Hekman, Joseph E. Kaserman, Jessie Huang, Konstantinos-Dionysios Alysandratos, Gabor E. Toth, Ferenc Jakab, Darrell N. Kotton, Andrew A. Wilson, Andrew Emili, Volker Thiel, John H. Connor, Gabor Kemenesi, Daniel Cifuentes, Elke Mühlberger

The x-axis label for panel D of Fig 5 is incorrect. The label should be: mAb114 concentration (μg/mL). The authors have provided a corrected version of Fig 5 here.

**Fig 5:**
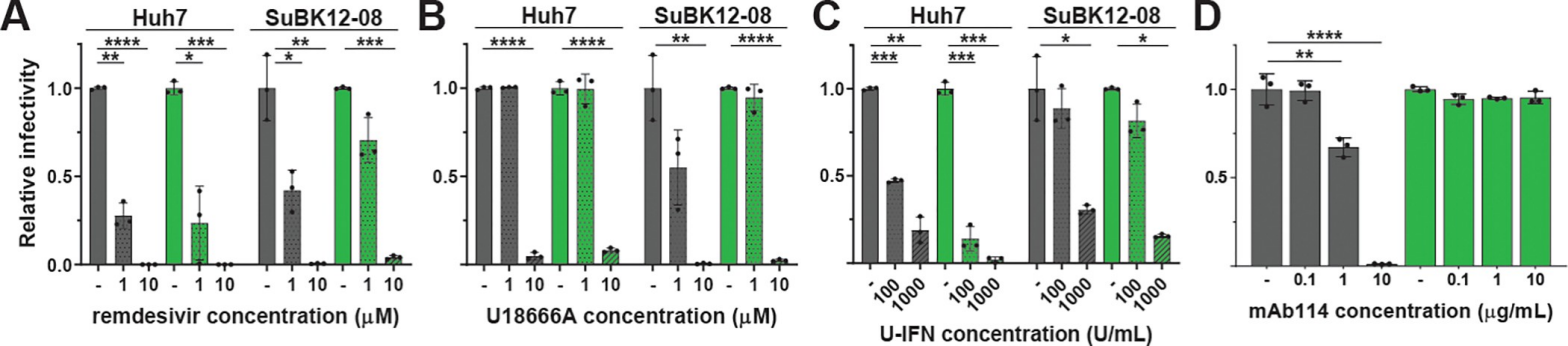
Antiviral testing of rLLOV_comp_. (A-D) Testing antiviral compounds against rEBOV-ZsG (gray bars) and rLLOV-ZsG (green bars) in human and bat cells. Huh7 and SuBK12-08 cells were pre-treated with the indicated concentrations of remdesivir (A) or the NPC-1 inhibitor U18666A (B) for 30 minutes, or with universal interferon (U-IFN) for 18 hours (C) prior to infection with rEBOV-ZsG or rLLOV-ZsG at an MOI of 0.1. Fluorescent images were taken at 2 dpi and mean fluorescence relative to infected cells pre-treated with vehicle control are shown. (D) Neutralization assay of rEBOV-ZsG and rLLOV-ZsG at an MOI of 10 using the indicated amounts of EBOV-neutralizing antibody mAb114. Fluorescent images were taken at 2 dpi and relative percentages of infected Huh7 cells are shown. Statistical differences were determined by two-way ANOVA (Prism), *p<0.05, **p<0.01, ***p<0.001, ****p<0.0001.
