# Supplementary figures and images for: Recombinant Lloviu virus as a tool to study viral replication and host responses
Source: PLoS Pathog. 2022 Feb 4;18(2):e1010268. doi: 10.1371/journal.ppat.1010268 (PMC8849519; doi:10.1371/journal.ppat.1010268)

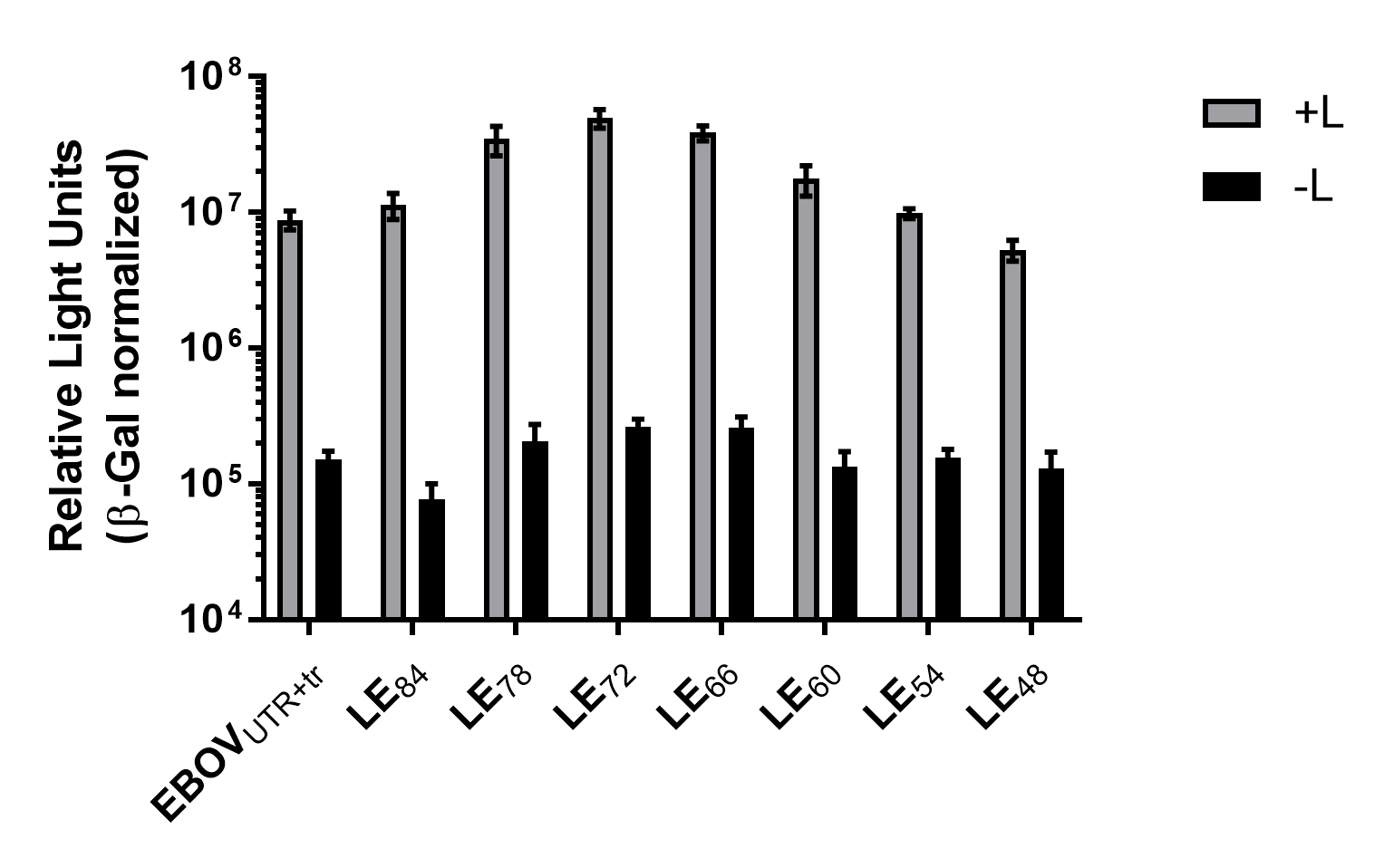

Supplement: S1 Fig — Mean firefly luciferase activity normalized to ß-galactosidase activity (transfection control) with standard deviation is shown. (TIF) [file ppat.1010268.s001.tif]

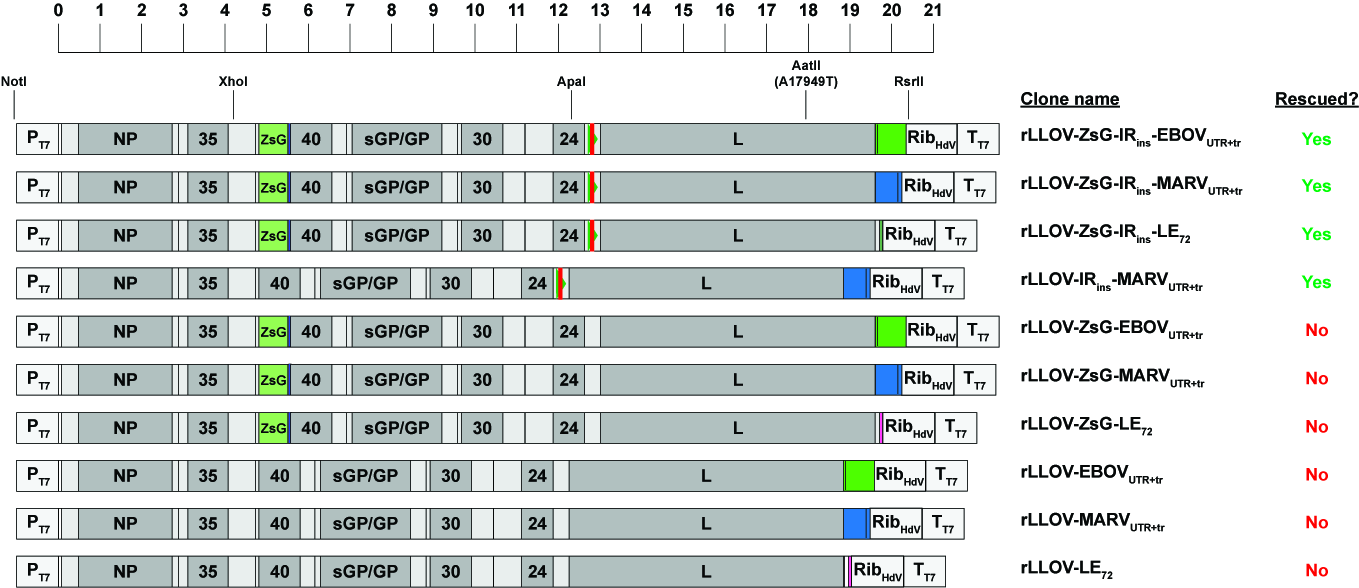

Supplement: S2 Fig — Schematics and names of full-length clones of rLLOVcomp that were rescued (above) and those that were not (below). Noncoding regions are indicated in light gray, LLOV genes are in gray, a ZsGreen-P2A reporter (ZsG) is in light green followed by a dark gray bar, gene start and end signals are black bars within noncoding regions, red bars indicate the IRins insertion in the VP24-L intergenic region, dark green indicates the Ebola virus (EBOV) L 3ˈ UTR and trailer (EBOVUTR+tr), dark blue indicates the Marburg virus L 3ˈ UTR and trailer (MARVUTR+tr), and pink indicates the last 72 nucleotides of the EBOV trailer (LE72). rLLOVcomp clones are to scale with the exception of the T7 RNA polymerase promoter (PT7), hepatitis delta virus ribozyme (RibHdV), and T7 RNA polymerase terminator sequences (TT7) which are enlarged for clarity. Scale bar above indicates 1 kb increments, starting with the first nucleotide of the rLLOVcomp clones. Important restriction enzymes that were used to clone full-length rLLOVcomp plasmids are indicated above, including an AatII site which introduces a silent mutation within L (A17949T for the rLLOV-ZsG-IRins-EBOVUTR+tr clone, corresponding to nucleotide 17164 of the published LLOV sequence, NCBI Reference Sequence NC_016144). (TIF) [file ppat.1010268.s002.tif]

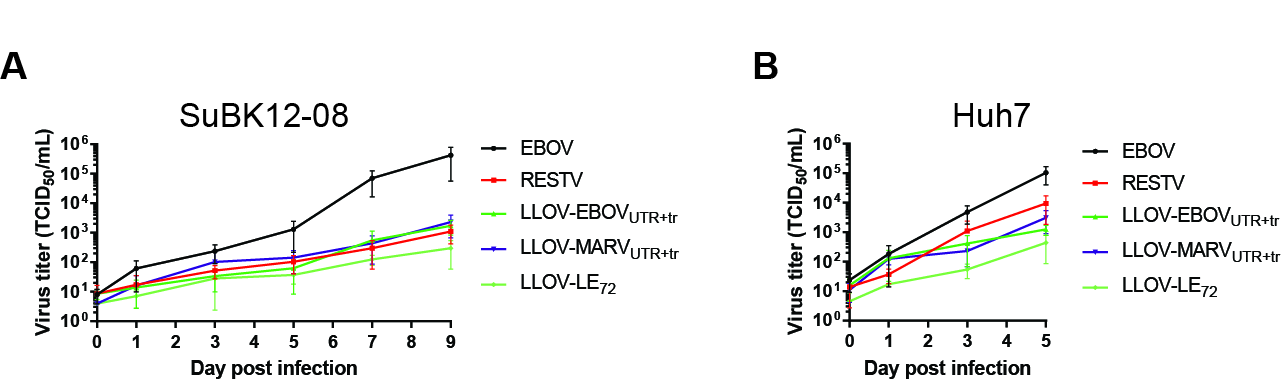

Supplement: S3 Fig — Growth curve of rEBOV, rRESTV, and the indicated versions of rLLOVcomp using an initial MOI of 0.01 in SuBK12 08 (A) and Huh7 cells (B). (TIF) [file ppat.1010268.s003.tif]

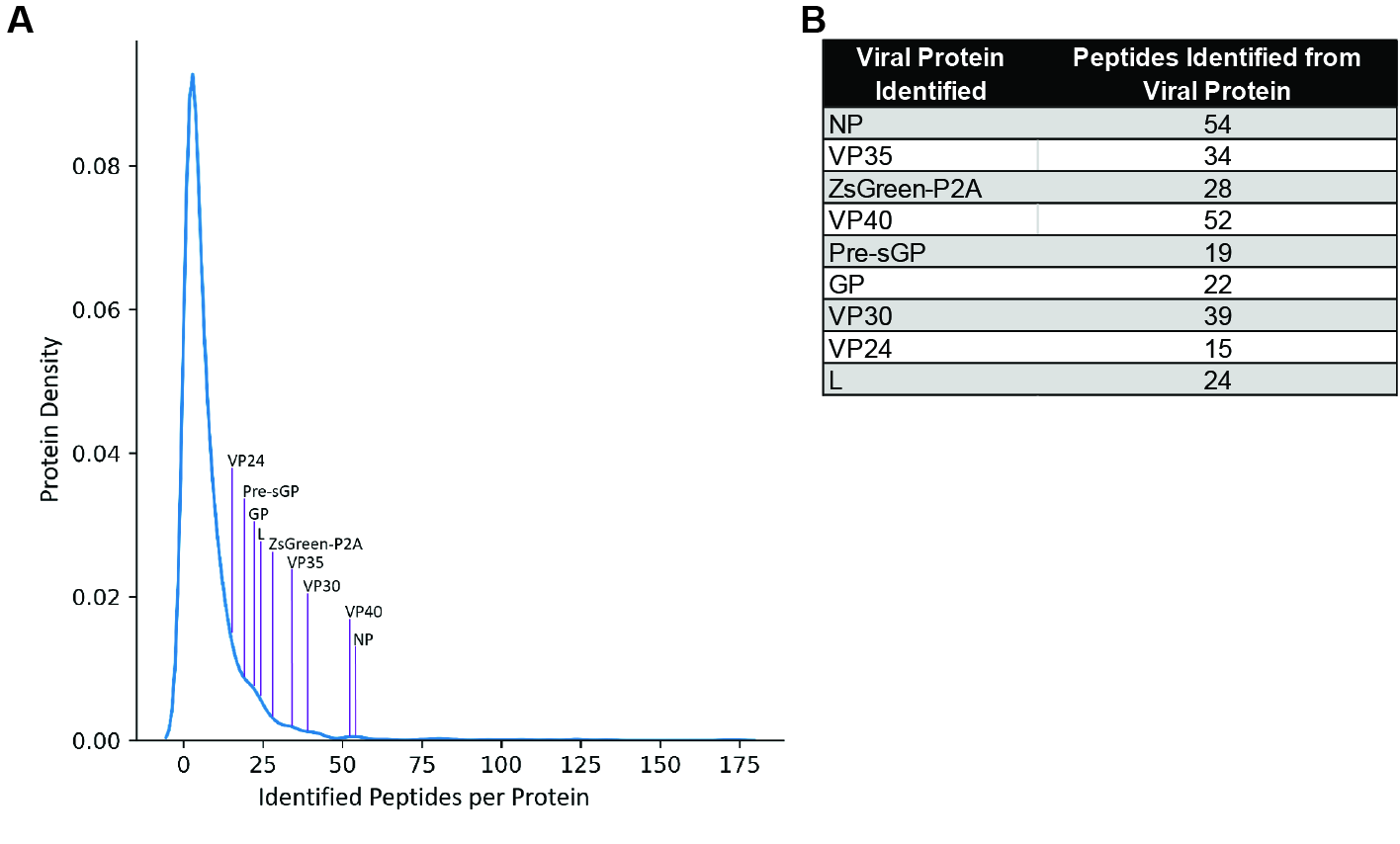

Supplement: S4 Fig — (A) Kernel density plot indicating the distribution of the number of identified peptides per protein across all 3,939 identified proteins within the rLLOVcomp-infected SuBK12-08 samples described in Fig 2E. The plot is annotated with the location of all viral proteins within the distribution. (B) Table of rLLOVcomp-encoded proteins and the number of peptides detected by mass spectrometry. (TIF) [file ppat.1010268.s004.tif]

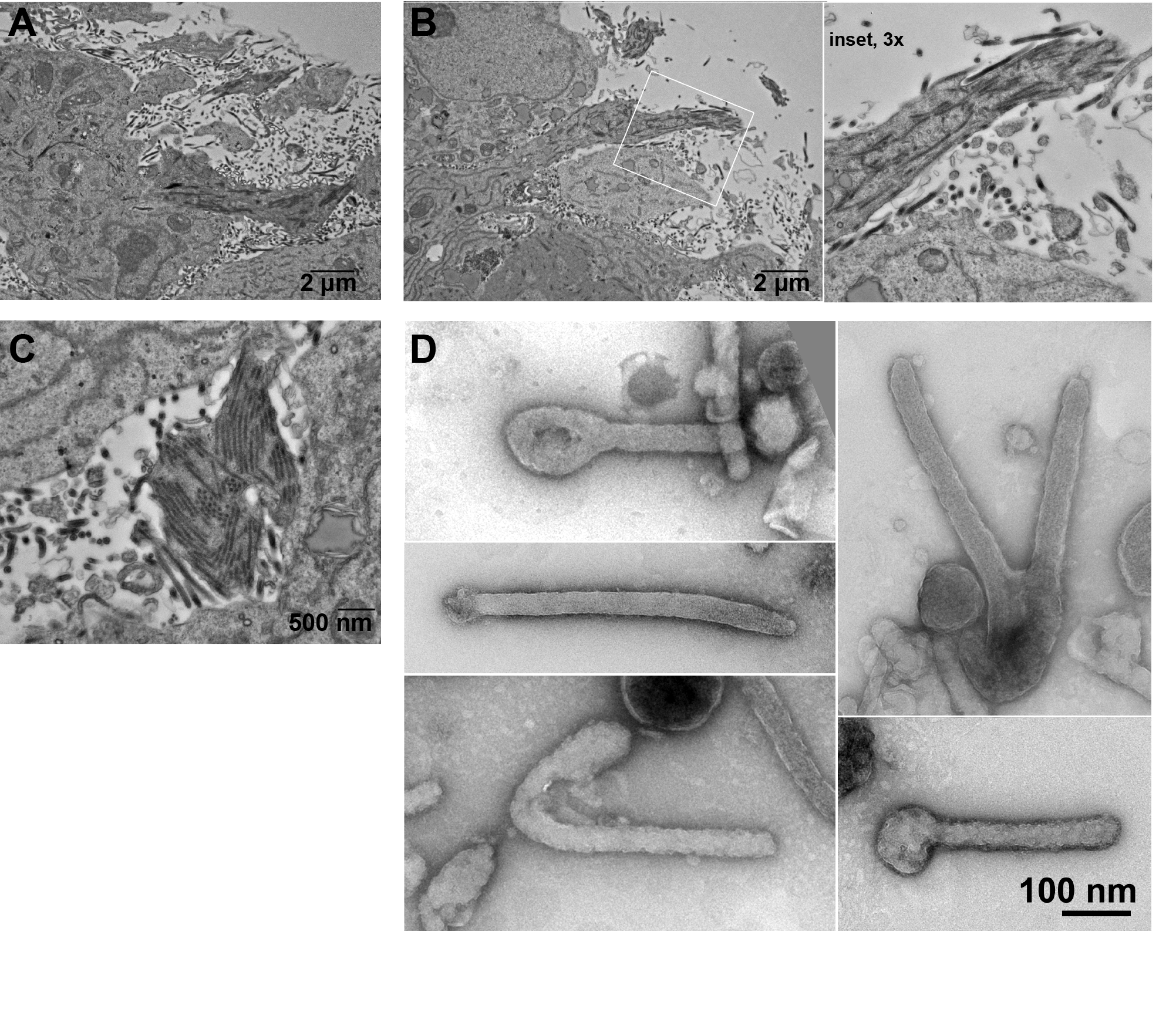

Supplement: S5 Fig — Huh7 cells were infected with EBOV at an MOI of 5 and fixed at 2 dpi. (A) Thin section of EBOV-infected cell releasing viral particles. (B) Thin section shows virus budding; inset shown on the right is magnified 3x; protein is black. (C) Circled area indicates EBOV inclusions containing cross sectioned and longitudinal sectioned nucleocapsids. (D) Negatively stained, isolated EBOV virions show heterogeneous ultrastructure, including 6-shaped, filamentous, shepherd’s hook, and branched filamentous structures, as seen previously [58]; protein is white. (TIF) [file ppat.1010268.s005.tif]

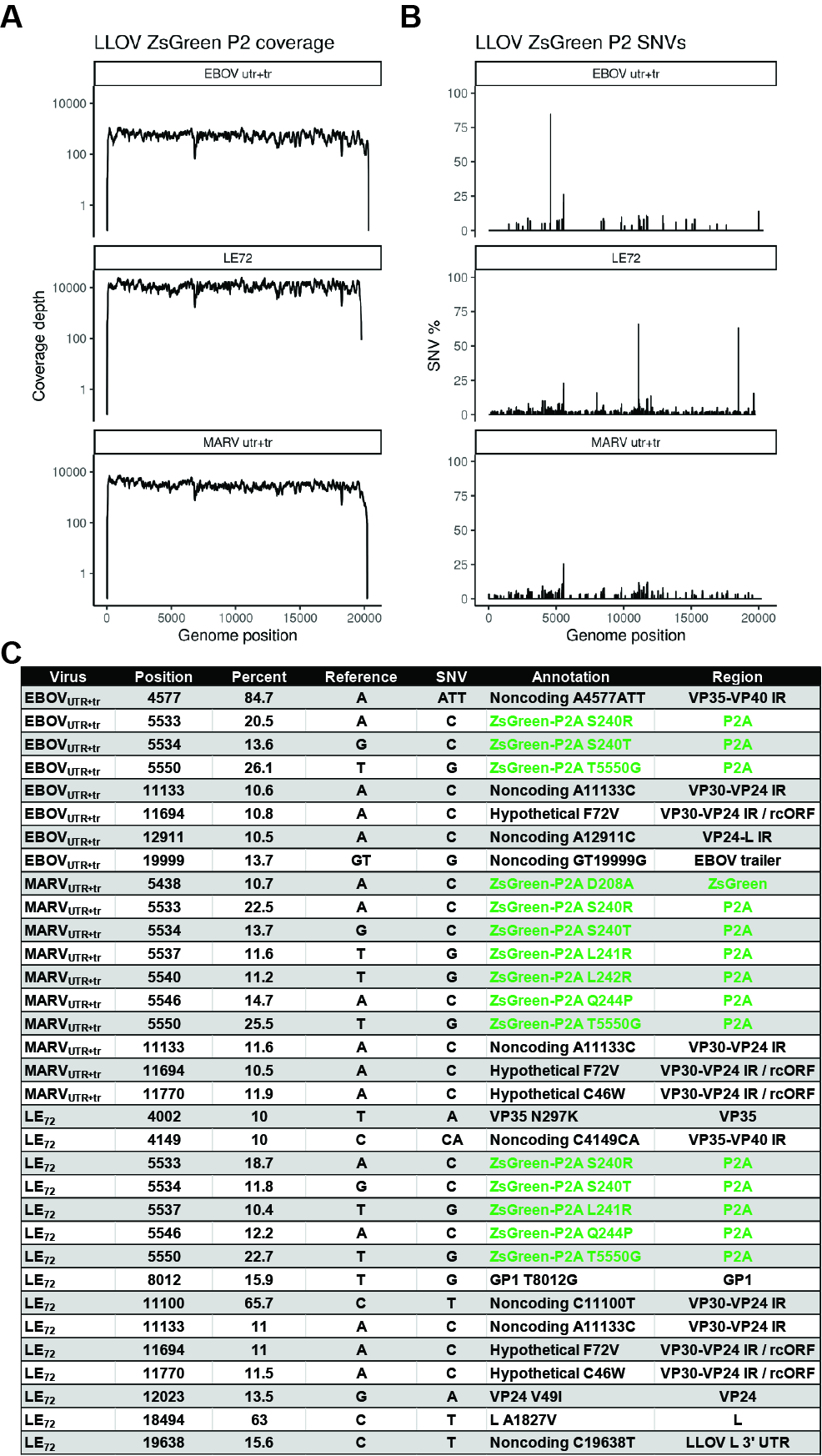

Supplement: S6 Fig — (A) Total RNA sequencing of passage 2 rLLOVcomp stocks yielded viral genome coverage ≥ 100x with the exception of the leader and trailer regions. Single nucleotide variable (SNV) plot (B) and Table (C), indicating the abundance and location within the genome of SNVs (SNVs ≥ 10% included in the Table). Few consensus SNVs (> 50%) were observed, whereas multiple subconsensus SNVs were observed at 10–20%. Mutations within the reporter gene (ZsGreen-P2A) are indicated in green. (TIF) [file ppat.1010268.s006.tif]

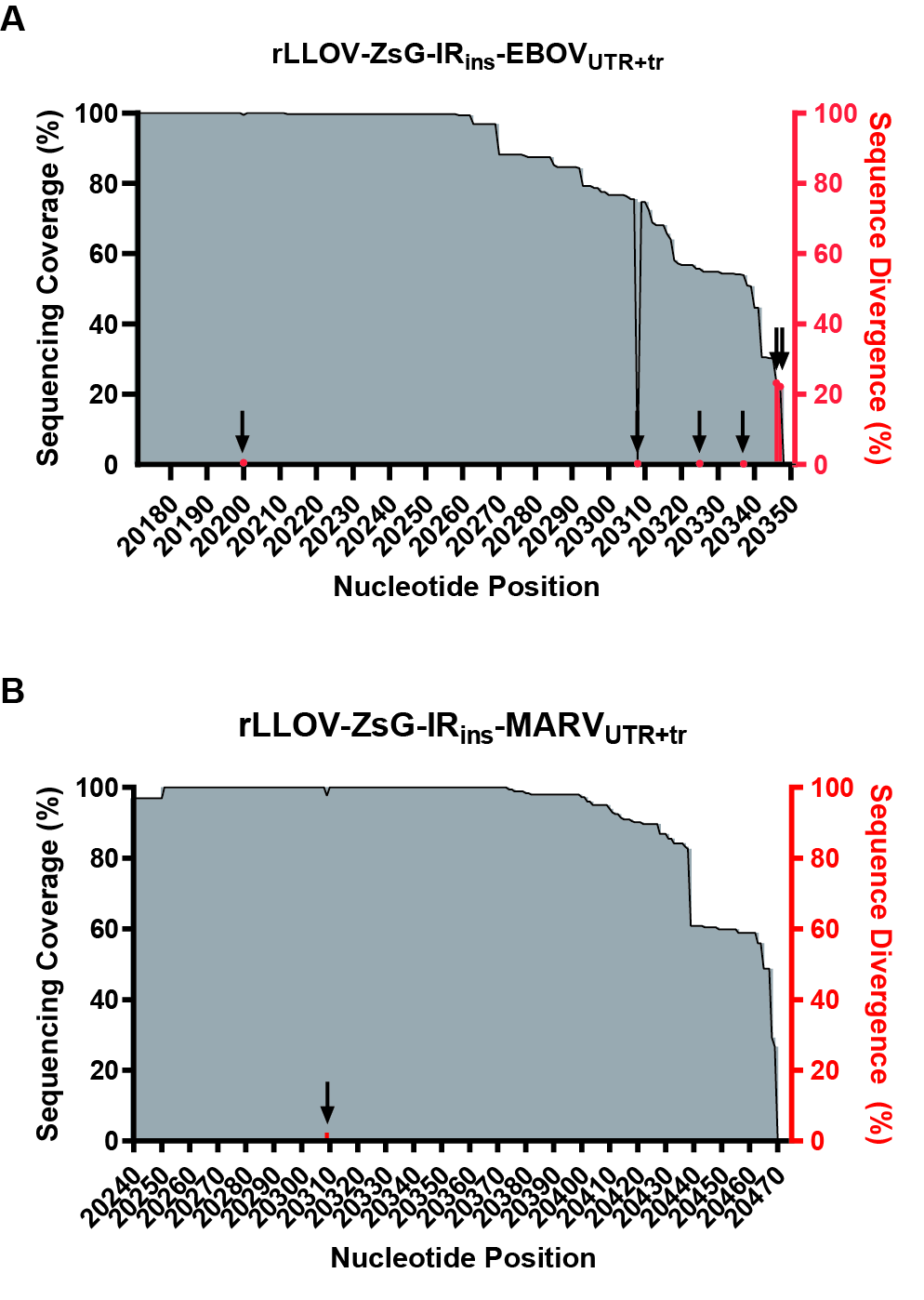

Supplement: S7 Fig — Sequence coverage of the trailer region of both (A) rLLOV-ZsG-IRins-EBOVUTR+tr and (B) rLLOV-ZsG-IRins-MARVUTR+tr virus stocks. Sequencing coverage percentages were calculated from the number of reads mapping to individual nucleotides within the respective viral genomes. Divergence from the EBOV trailer or MARV trailer sequences are plotted in red and highlighted with a black arrow. Sequencing results captured the terminal 175 nucleotides of the rLLOV-ZsG-IRins-EBOVUTR+tr sequence and the terminal 230 nucleotides of the rLLOV-ZsG-IRins-MARVUTR+tr sequence. Very few differences were observed between the rLLOVcomp full-length clone sequences and the viral stocks (passage 2). Very low abundance differences identified in the rLLOV-ZsG-IRins-EBOVUTR+tr stock include a deletion (position 20200, found in 0.58% of reads), an A insertion (between nucleotides 20308 and 20309, 0.33% of reads and the cause of the observed large dip in the graph due to its low abundance), a U to A mutation (position 20325, 0.3% of reads), and a U deletion (position 20337, 0.24% of reads) all in distinct, truncated species with very low read abundance. A significant portion of the reads (22.21% of reads) contained a terminal AC dinucleotide which is not present within the corresponding full-length clone. Similar to the data for the rLLOV-ZsG-IRins-EBOVUTR+tr stock, there are few differences noted between the rLLOV-ZsG-IRins-MARVUTR+tr sequence and corresponding rLLOVcomp full-length clone. The only low abundance difference observed was a single A deletion (position 20309, 2.35% of reads). There were no terminal nucleotide additions present in any of the rLLOV-ZsG-IRins-MARVUTR+tr sequence reads. (TIF) [file ppat.1010268.s007.tif]

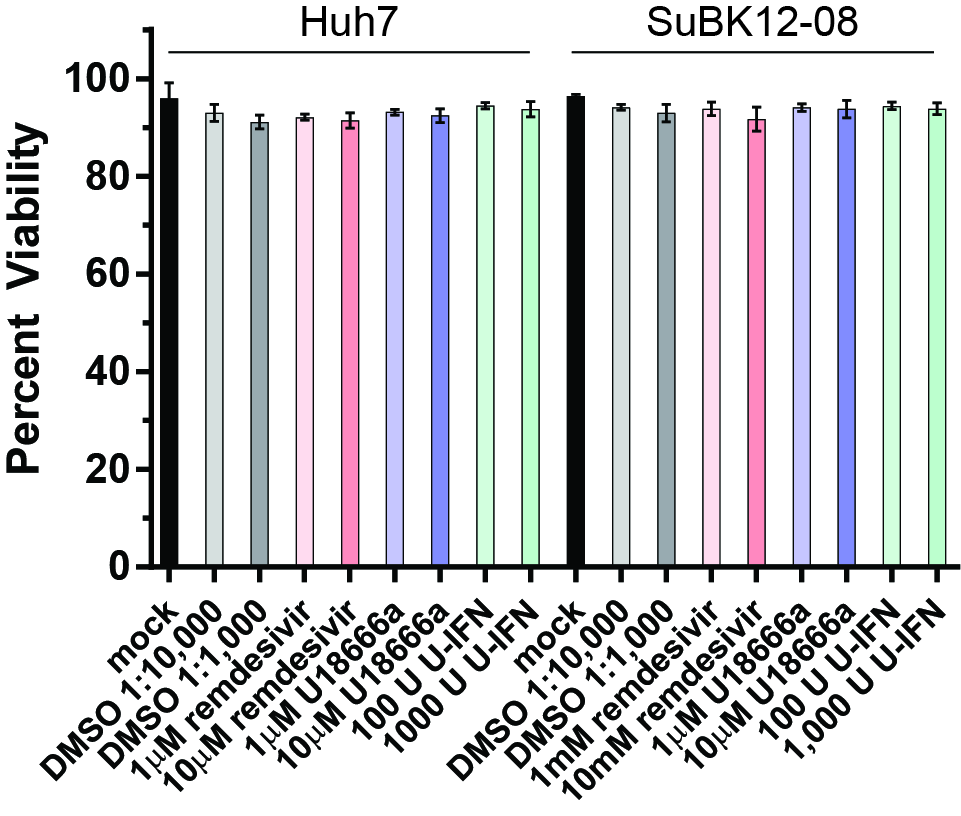

Supplement: S8 Fig — Huh7 and SuBK12-08 cells were treated as indicated for 2 days and cell viability was determined by trypan blue staining. (TIF) [file ppat.1010268.s008.tif]

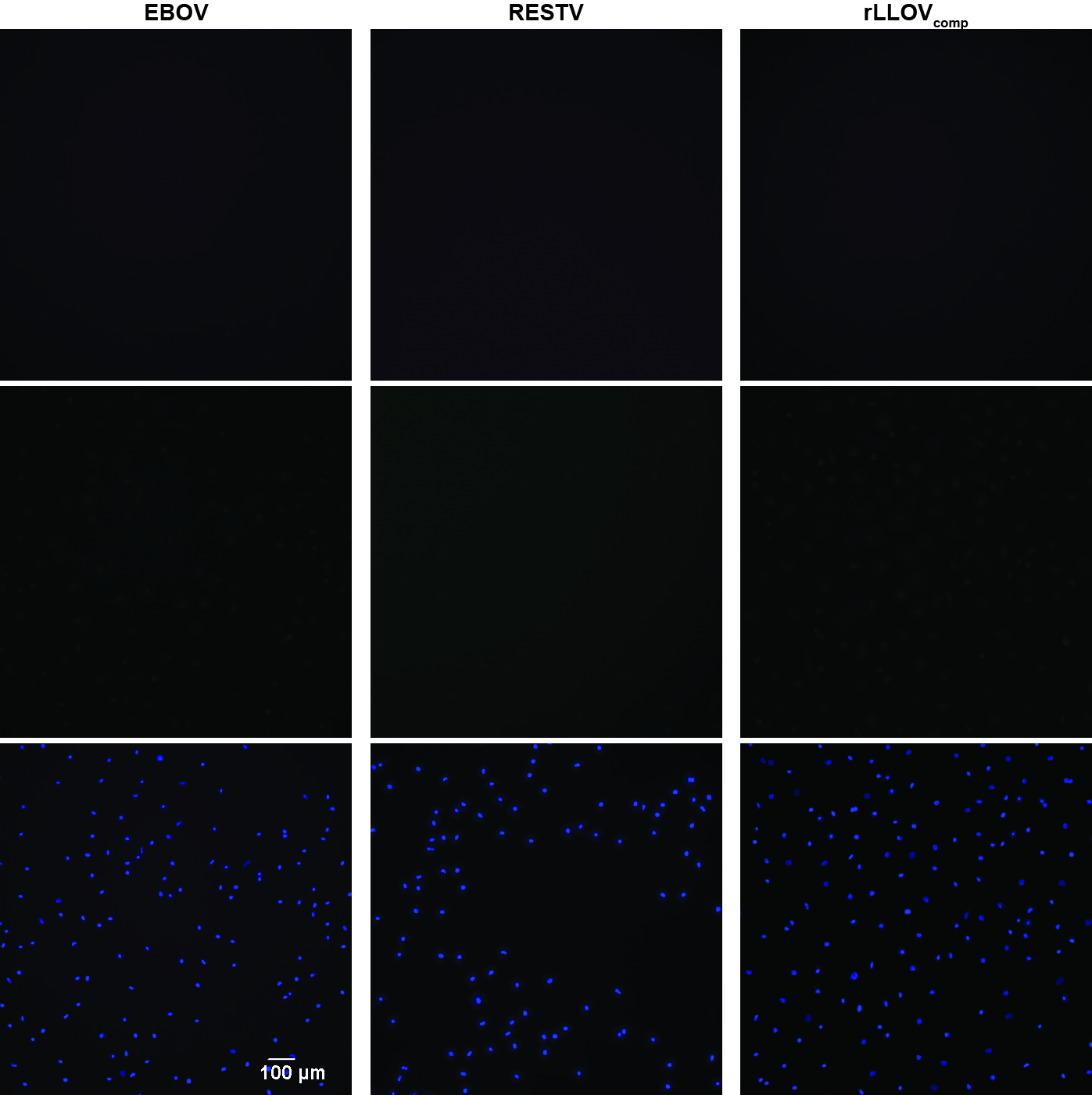

Supplement: S9 Fig — Human monocyte-derived macrophages (MDMs) were left uninfected, fixed at 1 dpi, and analyzed for the presence of negative (genome, magenta, in top row) and positive (mRNA and antigenome, green, in middle row) sense viral RNA using the indicated probe sets by RNA FISH. Cells were co-stained with DAPI (shown with merged channels, in bottom row). Indicated size bar is the same for all images. (TIF) [file ppat.1010268.s009.tif]

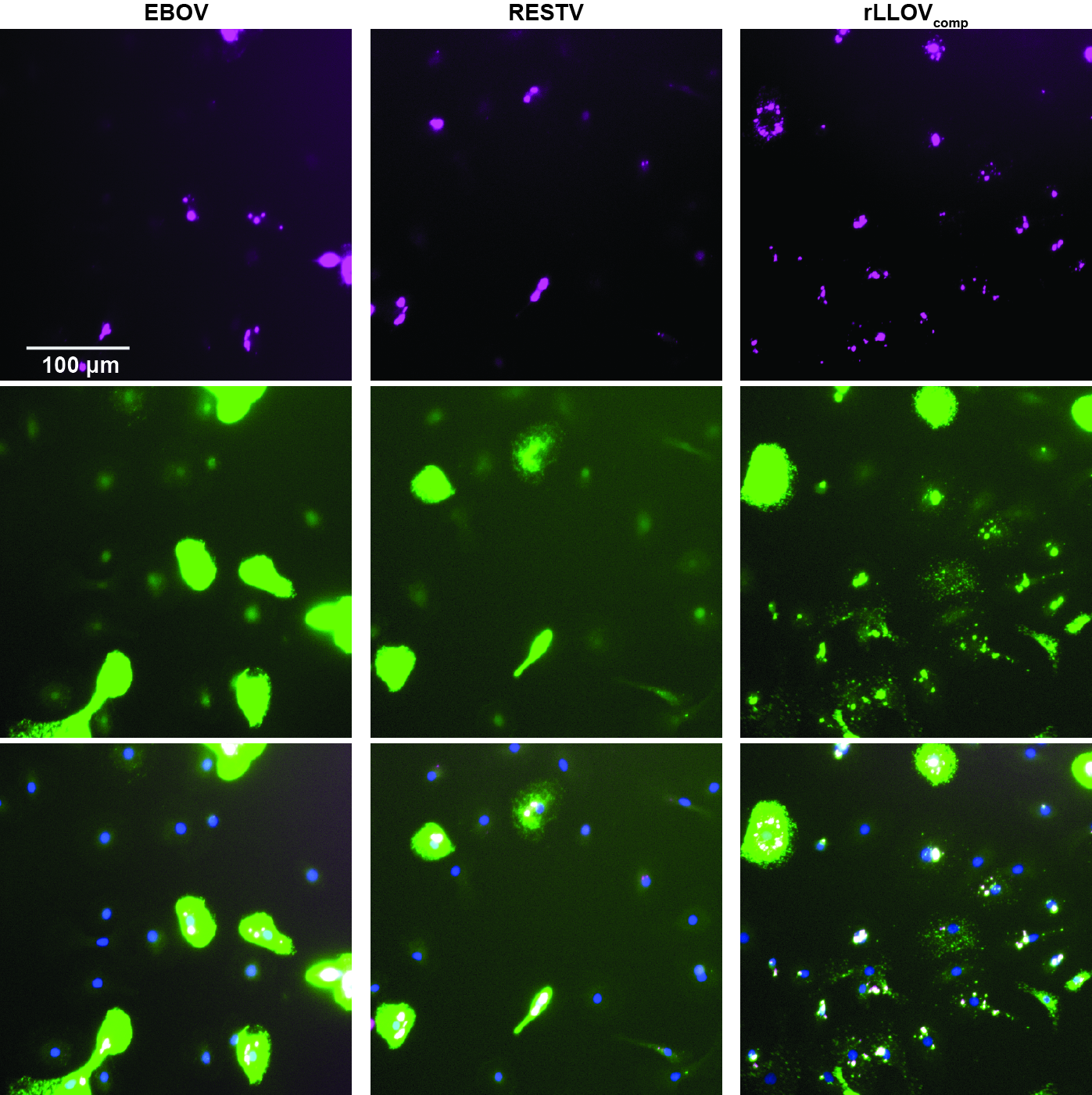

Supplement: S10 Fig — MDMs derived from donor 1 were infected at an MOI of 10 with EBOV, RESTV, or rLLOV-IRins-MARVUTR+tr. At 1 dpi, cells were fixed and analyzed for the presence of negative (genome, magenta, in top row) and positive (mRNA and antigenome, green, in middle row) sense viral RNA by RNA FISH. Cells were co-stained with DAPI (shown with merged channels, in bottom row). Images are shown with increased brightness in green and magenta channels to clearly visualize lower-intensity staining observed in rLLOV-IRins-MARVUTR+tr-infected cells. Indicated size bar is the same for all images. (TIF) [file ppat.1010268.s010.tif]
